# Value of Viral Nucleic Acid in Sputum and Feces and Specific IgM/IgG in Serum for the Diagnosis of Coronavirus Disease 2019

**DOI:** 10.3389/fcimb.2020.00445

**Published:** 2020-08-06

**Authors:** Yuwen He, Jiangyan Luo, Jie Yang, Jinlong Song, Li Wei, Weifeng Ma

**Affiliations:** ^1^Department of Pharmacy, The First Affiliated Hospital of Guangzhou Medical University, Guangzhou, China; ^2^Department of Microbiology, School of Public Health, Southern Medical University, Guangzhou, China; ^3^Department of Clinical Laboratory, The First Affiliated Hospital of Guangzhou Medical University, Guangzhou, China

**Keywords:** SARS-CoV-2, COVID-19, sputum, feces, nucleic acid test, serological test, IgM, IgG

## Abstract

A new type of coronavirus-induced pneumonia eventually termed “coronavirus disease 2019” (COVID-19) was diagnosed in patients in Wuhan (Hubei Province, China) in December 2019, and soon spread worldwide. To improve the detection rate of severe acute respiratory syndrome coronavirus 2 (SARS-CoV-2), we analyzed the results of viral nucleic acid and serum-specific antibody tests on clinical samples from 20 patients with SARS-CoV-2 infection diagnosed at the First Affiliated Hospital of Guangzhou Medical University in China. By comparing various sample types collected from COVID-19 patients, we revealed multiple pathways for SARS-CoV-2 shedding, and a prolonged detectable period for viral nucleic acid test in sputum specimens, demonstrating that the timeline of the viral shedding is of great value in determining the time of release from quarantine or discharge from hospital. We also recommend for the application of serological test to assist in confirming SARS-CoV-2 infection judged by viral nucleic acid test, especially when COVID-19-related symptoms have appeared and the viral nucleic acid test was negative. Our findings are critical for the diagnosis of SARS-CoV-2 infection and for determining deadline of restriction measures to prevent transmission caused by convalescent patients with COVID-19.

## Introduction

The severe acute respiratory syndrome coronavirus 2 (SARS-CoV-2), identified in Wuhan, China at the end of 2019, spread rapidly worldwide. In order to diagnose a large number of patients, samples of lower respiratory tract such as sputum with high positive rate are generally collected for viral nucleic acid detection (Han et al., 2020; Qu et al., 2020). In addition, some studies have reported the presence of viruses in feces (Tang et al., 2020; Wu et al., 2020), implying the risk of fecal-oral transmission, and indicating that specimen collection should not be limited to respiratory samples. Common symptoms at onset of illness were fever (98%), cough (76%), myalgia, or fatigue (44%) (Huang et al., 2020). However, it is worth noting that carriers who are subclinical can also infect people (Rothe et al., 2020). Therefore, it is necessary to deliver viral nucleic acid and serological tests for people with a history of exposure to SARS-CoV-2. We undertook a study on the viral nucleic acids of SARS-CoV-2 in swabs (nasal, pharyngeal), sputum and feces, as well as antibodies in the serum of COVID-19 patients admitted to the First Affiliated Hospital of Guangzhou Medical University, China. We aimed to clarify the importance of the test results of different specimen types for the diagnosis and de-isolation of patients with coronavirus disease 2019 (COVID-19).

## Methods

### Ethical Approval of the Study Protocol

The study protocol was approved by the Medical Ethics Committee of the First Affiliated Hospital of Guangzhou Medical University (Guangzhou, China). Written informed consent was obtained from all study participants.

### Collection and Processing of Specimens

From 29 January to 6 June 2020, samples (swabs, feces, sputum, and blood) of 20 COVID-19 patients were collected from the First Affiliated Hospital of Guangzhou Medical University, a designated hospital for the treatment of critically ill patients with COVID-19 in Guangzhou, a first-tier city in China. By the way, there were only 20 patients in this hospital who were almost critically ill or severe, other mild or asymptomatic patients were not admitted. The subjects of our study were all COVID-19 patients admitted to our hospital, and there were no additional inclusion or exclusion criteria. After admission, the patient was tested for viral nucleic acid continuously for a week and then every 2–3 days. Antibodies have been tested every 2–3 days since 15 February. To improve the efficiency of clinical work, collection of long-lasting negative specimens was suspended, while positive specimens were continuously collected until discharge. In the First Affiliated Hospital of Guangzhou Medical University, extraction of nucleic acids from the respiratory and fecal samples was performed with the commercialized nucleic acid extraction kits (Magnetic bead method, Daan Gene Co., Ltd. of Sun Yat-sen University, Guangzhou, China). The operations were completely following the instruction of the kit. The blood specimen was centrifuged with 3,000 rpm for 15 min to separate the serum within 24 h after collection, and then inactivated at 56°C for 30 min and stored at 4°C until use.

### RT-PCR

RNA was detected using the New Coronavirus 2019-nCoV Nucleic Acid Detection kit (Daan Gene Co., Ltd. of Sun Yat-sen University, Guangzhou, China). The analytical sensitivity of our kit was 500 copies/mL. This kit had no cross-reaction with other pathogens similar to SARS-CoV-2 or causing similar symptoms. Exogenous substances such as COVID-19 therapeutic drugs and endogenous substances such as blood and mucus in specimens did not interfere with the detection results of the kit. The primer and probe sequences designed for the open reading frame (ORF1ab), nucleoprotein (N) gene regions of SARS-CoV-2 are shown in Table 1. NC (ORF1ab/N) PCR reaction solution A and solution B were oscillated thoroughly after melting at room temperature followed by 8,000 rpm centrifugation for several seconds. Next, 17 μL of NC (ORF1ab/N) PCR reaction solution A and 3 μL of NC (ORF1ab/N) PCR reaction solution B were fully mixed and centrifuged briefly to bring down all the liquid to the bottom of the tube. Each PCR reaction tube was added with 20 μL of the above amplification system followed by adding 5 μL of the negative quality control (QC) substance, the viral nucleic acid of the sample to be tested, or positive QC substance. It was a one-step RT-PCR. The total PCR reaction volume was 25 μL. The volume of the template was 5 μL. The concentration of primers and probes was 1 μM and 10 μM in the final PCR reaction volume respectively. These PCR reaction tubes were centrifuged (8,000 rpm) for a few seconds at room temperature and placed in an RT-PCR instrument (ABI Prism 7500; Applied Biosystems, Foster City, CA, USA) for amplification and detection. The amplification process was as follows: 50°C for 15 min, and 95°C for 15 min, then 45 cycles were performed, including 94°C for 15 s, 55°C for 45 s, and the default melting curve steps of the RT-PCR instrument. If the Ct value of the tested sample was <40 in the FAM and VIC channels (FAM and VIC are fluorescent dyes), and there was an obvious amplification curve, the sample was judged to be positive for SARS-CoV-2.

**Table 1:** Primer and probe sequences.

**Genes**	**Primer sequences**		**Probe sequences**
	**Forward**	**Reverse**	
ORF1ab	CCCTGTGGGTTTTACACTTAA	ACGATTGTGCATCAGCTGA	5′-FAM-CCGTCTGCGGTATGTGGAAAGGTTATGG-BHQ1-3′
N	GGGGAACTTCTCCTGCTAGAAT	CAGACATTTTGCTCTCAAGCTG	5′-FAM-TTGCTGCTGCTTGACAGATT-TAMRA-3′

### Serological Test

Our serological test used for SARS-CoV-2 specific IgM/IgG antibodies was a rapid detection method. The detection principle of the IgM/IgG antibody detection kit (Livzon Diagnostics Co., Ltd, Zhuhai, China) for SARS-CoV-2 is based on colloidal gold immunochromatography. The antibody detection kit was proved that there was no cross-reaction to the kit in the detection of IgM/IgG-positive samples of similar or other viruses. It has been experimentally verified that a variety of exogenous and endogenous substances have not interfered with our antibody detection kit, and the presence of the SARS-CoV-2 IgG antibody does not affect the detection of SARS-CoV-2 IgM antibody and vice versa. The clinical test results of this kit showed that the detection sensitivity of IgM was 79.0% and the specificity was 99.7%; the detection sensitivity of IgG was 84.3% and the specificity was 99.4%; the combined detection sensitivity of IgM and IgG was 90.6% and the specificity was 99.2%. Serum (10 μL) was added to the sample well of the IgM and IgG detection cards. Then, two drops (~100 μL) of sample diluent were added vertically. If the detection line and QC line appeared within 15 min, then the sample was judged to be positive.

### Statistical Analyses

Data processing was carried out using SPSS v22.0 (IBM, Armonk, NY, USA). The differences between samples or individuals were analyzed by ANOVA of randomized block design data and Bonferroni multiple comparison. Data are presented as mean ± SD. Time from onset to admission and hospital stay are presented as median (IQR). ANOVA of randomized block design data with 2-sided *P* < 0.05 was considered significant. Multiple comparison with 2-sided *P* < 0.0083 was considered significant.

## Results

### Characteristics of Cases

**Figure 1:**
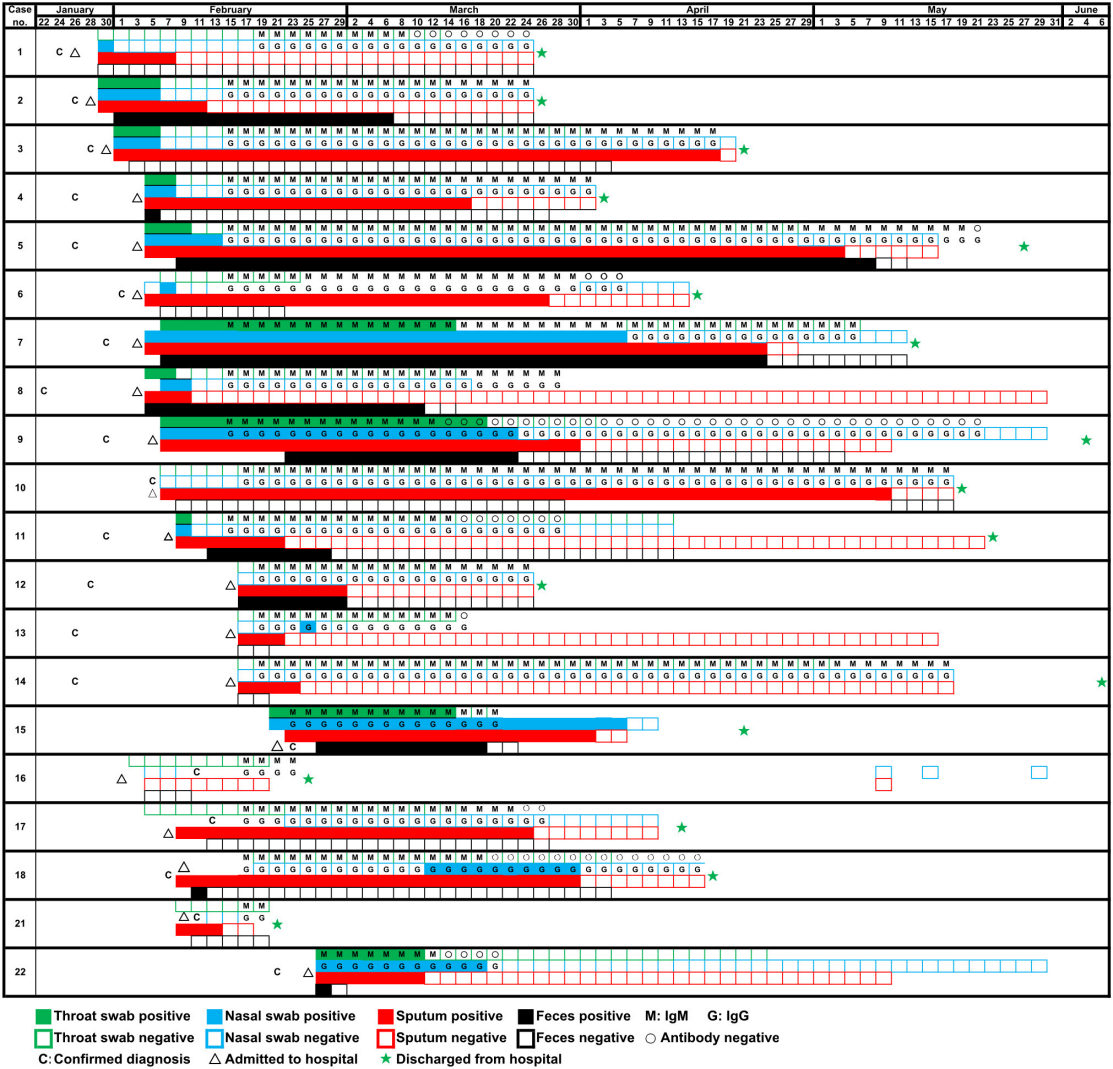
Detection of SARS-CoV-2 RNA of four types of samples (throat swab, nasal swab, sputum, and feces) and specific antibody of 20 patients on different dates.

A total of 22 patients were diagnosed as COVID-19 in our hospital, of which two (patients #19, #20) were transferred the next day after hospital admission. The median time from onset to admission to our hospital for 20 patients was 9.5 (7.5–14.0), some patients had been treated elsewhere during this period. And the median hospital stay was 63.5 (28.0–93.8) days. In fact, a longer time from onset to admission may result in more serious symptoms or organic damages, thereby increasing the difficulty of clinical cure (Qi et al., 2020). Of 20 patients with COVID-19, the mean age were 57.35 years old. The ratio of males: females was 14:6. According to the clinical diagnosis, the ratio of patients with critical: severe: mild disease was 15:2:3. The clinical characteristics were summarized in Table 2. During hospitalization, the most common symptoms of 20 patients were fever (20 [100%]), cough (17 [85%]), shortness of breath (15 [75%]), sputum production (13 [65%]), and fatigue (10 [50%]); less common symptoms were headache (3 [15%]) and diarrhea (2 [10%]). In addition, 17 (85%) patients had comorbidities, including 14 (70%) acute respiratory distress syndrome, 9 (45%) myocardial damage, and 8 (40%) hypertension, etc. Specimens were collected from these 20 patients admitted to the hospital since January 29. As of May 10, all 20 patients had turned negative for viral nucleic acid. The discharge criteria include: (i) Body temperature returned to normal for more than 3 days; (ii) Respiratory symptoms were significantly improved; (iii) Chest images showed that acute exudative lesions were significantly improved; (iv) Viral nucleic acid tests were negative in sputum, nasopharyngeal swabs, and feces samples for two consecutive times (at intervals of more than 24 h). Seventeen patients had been discharged from the hospital on June 7, while three patients (patients #8, #13, #22) had not been discharged so far because of other underlying diseases. Among the 20 tested patients, the time-dependent diagnostic results of SARS-CoV-2 RNA in throat swabs, nasal swabs, sputum, and feces were summarized in Figure 1, as well as serum specific IgM/IgG antibodies. When all types of specimens turned negative, the symptoms of most patients had completely disappeared or were alleviated.

**Table 2:** Clinical characteristics of patients infected with COVID-19.

	**Patients (*n* = 20)**
**Signs and symptoms during hospitalization**	
Fever	20 (100%)
Cough	17 (85%)
Shortness of breath	15 (75%)
Sputum production	13 (65%)
Fatigue	10 (50%)
Headache	3 (15%)
Diarrhea	2 (10%)
**Comorbidities**	
Acute respiratory distress syndrome	14 (70%)
Myocardial damage	9 (45%)
Hypertension	8 (40%)
Respiratory failure	8 (40%)
Sepsis	6 (30%)
Diabetes	6 (30%)
Kidney disease	5 (25%)
Dysfunction or abnormal blood coagulation	4 (20%)
Shock	4 (20%)
Chronic liver disease	4 (20%)
Chronic lung disease	3 (15%)

*Data are n (%)*.

### High Detection Rate and a Long Positive Duration of SARS-CoV-2 in Sputum Samples

**Figure 2:**
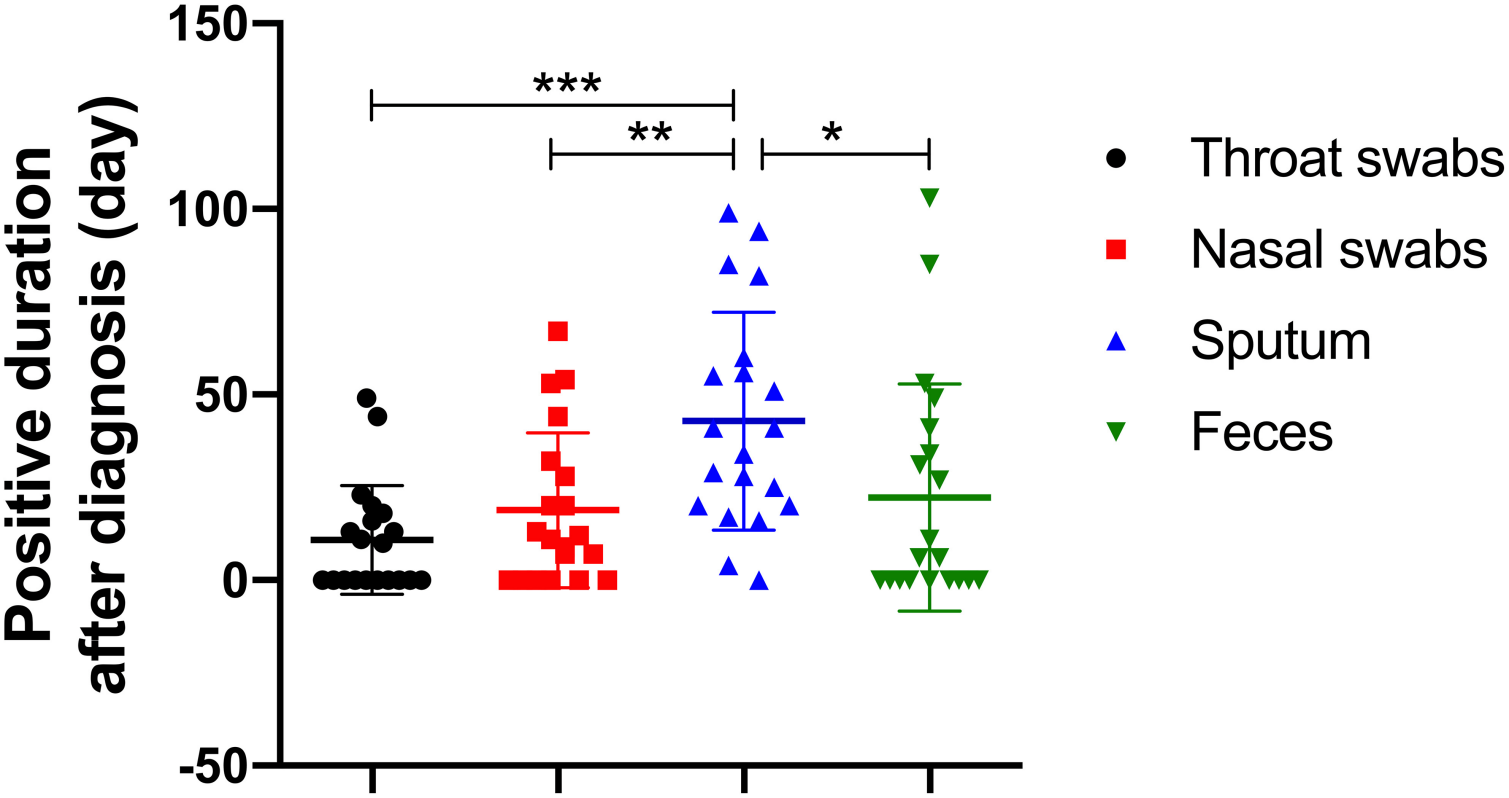
The number of days it took for SARS-CoV-2 RNA to shed in four types of sample (throat swab, nasal swab, sputum, and feces). *p < 0.00833, **p < 0.00167, ***p < 0.00017.

Sputum samples were positive in 19 of the 20 (95%) patients with COVID-19. In other words, of all the sample types tested, the detection rate of the viral nucleic acids of SARS-CoV-2 was highest in sputum samples. Interestingly, SARS-CoV-2 nucleic acid tests in nasal swabs, throat swabs and feces for a small number of patients (patients #10, #14, #17, and #21) were invariably negative from diagnosis to discharge but lasted for 94, 29, 41, and 4 days in sputum, respectively. Moreover, sputum samples remained positive for an average of 42.8 ± 4.2 (mean ± SD) days since diagnosis. The comparison of the virus-carrying duration showed that the persistence of SARS-CoV-2 RNA in sputum stayed significantly longer than that in throat swabs, nasal swabs, and feces (Figure 2), which prolonged by 32.0, 24.0, and 20.6 days, respectively. Among them, patient #5 even continued to be positive for 99 days. In short, sputum has a high detection rate of viral nucleic acid and a long-term continuous positive.

### Prolonged Presence of SARS-CoV-2 in a Part of Fecal Samples

The feces samples remained SARS-CoV-2 RNA positive for 22.3 ± 29.8 (mean ± SD) days since diagnosis, of which 11 patients (55%) were positive. Although the existence time of virus in feces was not significantly different from that in nasal or throat swabs (Figure 2), it is worth noting that patients #2 and #8 presented consecutively positive in feces samples for 24.0 and 29.0 days, respectively after the nasal-swab, throat-swab, and sputum samples consistently became negative. Moreover, patients #5 with the longest positive duration in the sputum had a longer feces duration, which took 103 days before it turned negative.

### High Sensitivity of Serological Detection

Although the detection of virus nucleic acid based on RT-PCR has high sensitivity, it is inevitable to produce false-negative results according to our experience and other references (Li et al., 2020). The potential problem of a high false-negative rate caused by the nucleic-acid test prompted us to increase the detection of IgM and IgG antibodies against SARS-CoV-2 from 15 February 2020. These specific antibodies against SARS-CoV-2 were successfully detected in all of 20 patients (100%) (Figure 1), with the exception of two patients (patients #19, #20) who were not tested. Otherwise, a few cases such as patient #16 would likely be considered to be SARS-CoV-2-negative through routine RNA testing and, thus, pose a threat to other people. Initially, we observed that patient #16 presented fever and diarrhea, and chest X-ray/CT showed unilateral pulmonary infection. Epidemiological investigation showed a history of passing through the epidemic area and similar symptoms appear on her spouse. For this patient, SARS-CoV-2 RNAs in all kinds of samples were negative while SARS-CoV-2 IgG and IgM remained positive. In view of the above circumstances, we finally took IgM/IgG positive as the diagnostic criteria to include this patient. In a word, we found that the combined detection of viral nucleic acid and specific antibody successfully improved the detection rate of SARS-CoV-2.

## Discussion

In this study, we examined the clinical biological samples of all COVID-19 patients in the first affiliated Hospital of Guangzhou Medical University, most of them were critical or severe patients. Compared with mild patients (Wölfel et al., 2020), our specimens usually have a longer time for virus shedding, and critically ill patients have a longer period of treatment and observation, which provides a reference for the discharge and isolation time of critically ill patients. Secondly, since COVID-19 was first prevalent in China, we successfully demonstrated the whole process from diagnosis to discharge, while other areas may have not detected until the endpoints of some critically ill patients from the beginning of the epidemic to the present. One limitation is that the sample size is relatively small, and more data on critically ill patients need to be collected in the future to further confirm our results. We also call for a follow-up of discharged patients to observe the prognosis or re-positive conditions, although we failed to collect these useful data in time.

Our results show that viral nucleic acid has a high detection rate and a significantly long existence time in the sputum from COVID-19 patients. Research by Qu et al. (2020) also showed that SARS-CoV-2 RNA is still detectable in sputum obtained by atomization from cured patients, although the pharynx swab test is negative. In addition, the viral load in the sputum is the highest compared to other specimens in the later stage of COVID-19 (Yoon et al., 2020). Therefore, it is necessary to collect lower respiratory tract specimens for SARS-CoV-2 RNA testing before COVID-19 patients can be discharged. Taken together, these results indicate that the sputum test was a more reliable criterion for discharge from hospital or quarantine release testing from a specimen taken from the upper airways. Moreover, since some patients (10%, such as patients #2 and #8) have positive feces longer than sputum, we recommend that the combined detection of feces and sputum is more reliable. Our data suggest that the presence of SARS-CoV-2 RNA in feces may be prolonged for more than a month in exceptional cases after a negative result in nasal-swab, throat-swab, and sputum samples. This observation is similar to that of Wu et al. (2020), who found that the positivity of SARS-CoV-2 RNA in fecal samples lagged behind that in respiratory samples. Currently, although no cases of SARS-CoV-2 transmission through fecal-oral route have been reported, the potential risk of fecal-oral transmission may be increased in closed residential spaces and areas with poor sanitary conditions. For themselves, SARS-CoV-2 can actively replicate in human intestinal organs, and infectious viruses can be isolated from fecal samples of COVID-19 patients (Zhou et al., 2020). Our results suggested that infected patients could potentially shed SARS-CoV-2 through respiratory and fecal-oral routes. Therefore, we suggest that RT-PCR should be employed to routine diagnosis of fecal samples after removal of SARS-CoV-2 RNA from the respiratory tract. A negative fecal result for SARS-CoV-2 nucleic acids in feces could be included in the rules for discharge from the hospital or lifting of quarantine measures as a supplement to sputum detection for patients recovering from COVID-19.

However, a negative result of viral nucleic acid cannot rule out the possibility of SARS-CoV-2 infection. We have further confirmed that combined detection of serum SARS-CoV-2 IgM and IgG was a practical and sensitive indicator, and also an effective complement to viral nucleic acid testing considering false-negative results upon it (Li et al., 2020). In addition, the duration of IgM/IgG-positivity from serum samples was much longer than that of SARS-CoV-2 nucleic acids from clinical samples. Serological test appears to be more meaningful for patients with an exposure history but who are negative for SARS-CoV-2 nucleic acids, regardless of whether the patients present symptoms or not. We speculate that serological test can effectively make up for the omission risk of viral nucleic acid detection, thus possessing important value in the timely diagnosis and prevention of COVID-19. Although nucleic acid test is difficult to avoid false negative, it can directly detect whether there is SARS-CoV-2 virus in human body, and its positive result is of great significance and is the gold standard for COVID-19 diagnosis (Shen et al., 2020). Compared with serological detection, nucleic acid detection can be applied in the early stage of SARS-CoV-2 infection, and indicate that it is now undergoing an infected state. However, in view of the high sensitivity of specific antibody detection, we suggest that it can be used as a supplementary indicator for nucleic acid detection, or even as a targeted remedy for leakage.

In summary, SARS-CoV-2 has multiple shedding ways and a more prolonged survival period in sputum specimens from COVID-19 patients. A comprehensive understanding of the viral shedding period in human body is extremely helpful to determine the time of release from quarantine or discharge from the hospital. We also recommend the application of a serological test to assist in identifying SARS-CoV-2 infection judged by viral nucleic acid test, especially when COVID-19-related symptoms have presented. Since COVID-19 has progress to one of the latest severe infectious diseases threatening human and restricting social activities worldwide, our findings are critical for the diagnosis of SARS-CoV-2 infection and the prevention of virus transmission in convalescent COVID-19 patients.

## Data Availability Statement

All datasets generated for this study are included in the article/supplementary Material.

## Ethics Statement

The studies involving human participants were reviewed and approved by the First Affiliated Hospital of Guangzhou Medical University (Guangzhou, China). The patients/participants provided their written informed consent to participate in this study. Written informed consent was obtained from the individual(s) for the publication of any potentially identifiable images or data included in this article.

## Author Contributions

WM and LW conceived and designed the study. JS conducted the experiments. JL analyzed the data. YH and JY wrote the paper. All authors reviewed and edited the manuscript. All authors contributed to the article and approved the submitted version.

## Conflict of Interest

The authors declare that the research was conducted in the absence of any commercial or financial relationships that could be construed as a potential conflict of interest.
